# Visual hallucinations in Alzheimer's disease do not seem to be associated with chronic hypoperfusion of to visual processing areas V2 and V3 but may be associated with reduced cholinergic input to these areas

**DOI:** 10.1186/s13195-019-0519-7

**Published:** 2019-09-12

**Authors:** Lindsey Isla Sinclair, Amit Kumar, Taher Darreh-Shori, Seth Love

**Affiliations:** 1 Population Health Sciences, Oakfield House, University of Bristol, Clifton, Bristol, BS8 2BN UK; 2 Translational Health Sciences, Level 1 Learning & Research Building, Southmead Hospital, University of Bristol, Bristol, BS10 5NB UK; 3 Division of Clinical Geriatrics, NEO Plan 7, Department of Neurobiology, Care Sciences and Society (NVS), H1, 141 52 Huddinge, Sweden

**Keywords:** Dementia, Alzheimer’s disease, Visual hallucinations, Vascular endothelial growth factor, Myelin-associated glycoprotein, PLP1, von Willebrand factor, Acetylcholine, Post-mortem tissue

## Abstract

**Background:**

Up to 20% of patients with AD experience hallucinations. The pathological substrate is not known. Visual hallucinations (VH) are more common in dementia with Lewy bodies (DLB). In autopsy studies, up to 60% of patients with AD have concomitant Lewy body pathology. Decreased perfusion of the occipital lobe has been implicated in DLB patients with VH, and post-mortem studies point to both decreased cholinergic activity and reduced oxygenation of the occipital cortex in DLB.

**Methods:**

We used biochemical methods to assess microvessel density (level of von Willebrand factor, a marker of endothelial cell content), ante-mortem oxygenation (vascular endothelial growth factor, a marker of tissue hypoxia; myelin-associated glycoprotein to proteolipid protein-1 ratio, a measure of tissue oxygenation relative to metabolic demand), cholinergic innervation (acetylcholinesterase and choline acetyltransferase), butyrylcholinesterase and insoluble α-synuclein content in the BA18 and BA19 occipital cortex obtained post-mortem from 23 AD patients who had experienced visual hallucinations, 19 AD patients without hallucinations, 19 DLB patients, and 36 controls. The cohorts were matched for age, gender and post-mortem interval.

**Results:**

There was no evidence of reduced microvessel density, hypoperfusion or reduction in ChAT activity in AD with visual hallucinations. Acetylcholinesterase activity was reduced in both BA18 and BA19, in all 3 dementia groups, and the concentration was also reduced in BA19 in the DLB and AD without visual hallucinations groups. Insoluble α-synuclein was raised in the DLB group in both areas but not in AD either with or without visual hallucinations.

**Conclusions:**

Our results suggest that visual hallucinations in AD are associated with cholinergic denervation rather than chronic hypoperfusion or α-synuclein accumulation in visual processing areas of the occipital cortex.

**Electronic supplementary material:**

The online version of this article (10.1186/s13195-019-0519-7) contains supplementary material, which is available to authorized users.

## Introduction

Alzheimer’s disease (AD) is the most common form of late-life dementia. Its incidence is set to nearly triple by 2050, due to the ageing of the population [1]. As with all dementias, it has a devastating effect on patients and those around them. The pathological hallmarks of AD are neurofibrillary tangles and amyloid-β plaques. Up to 20% of patients with AD experience hallucinations during their illness [2]. Current treatments for this can be ineffective, and antipsychotics are often used, which increase the risk of stroke and premature death [3].

Visual hallucinations can be very distressing for both patients and their relatives. They occur in most forms of dementia but are particularly common in dementia with Lewy bodies (DLB) [4]. The core pathology in DLB is the accumulation of α-synuclein in Lewy bodies and Lewy neurites [5]. In autopsy studies, up to 60% of patients with AD had at least some concomitant Lewy body pathology [4]. Several studies have found that visual hallucinations are more likely in those with AD and Lewy body pathology [6–9]. One large study suggested that there was an AD plus Lewy body phenotype consisting of dementia with delusions, hallucinations and prominent motor problems [10].

Most of the work on the pathophysiology of visual hallucinations in dementia has been in DLB. Neuroimaging studies have shown decreased perfusion of the occipital lobe, a region of the cortex with only sparse Lewy bodies [11–13]. A PET study found reduced fluorodeoxyglucose uptake in the occipital cortex of individuals with DLB who had visual hallucinations [14]. A SPECT study which compared DLB to AD found reduced perfusion in the medial occipital lobe in DLB but not in AD [15]. Indeed reduced occipital lobe perfusion is now included as a supportive biomarker for the diagnosis of DLB [16]. Post-mortem studies have suggested decreased cholinergic activity in the visual cortex in DLB [17] and decreased oxygenation of the occipital cortex in DLB, which correlated with reduced microvessel density and was associated with decreased VEGF [18].

Acetylcholinesterase inhibitors such as donepezil have been shown to reduce visual hallucinations in DLB, although they do not help all patients [19]. A small uncontrolled study showed that donepezil increased occipital lobe perfusion on SPECT and decreased hallucinations [20]. Work in myocytes has suggested an intriguing direct link between cholinergic stimulation and VEGF level [21]. It is also known that cholinergic stimulation causes vasodilatation [22].

Current theories of the basis of visual hallucinations in dementia include the perception and attention deficit model, which proposes a lack of integration of incoming sensory information and top-down prior knowledge/expectation [23]. It has advantages over other models, e.g. the misidentification model, in that it better accounts all of the observed phenomena in different disease states. This model implicates the ventral visual stream in the recurrent complex visual hallucinations seen in DLB as this stream is more involved in object-based attention. It has been suggested that cholinergic input modulates the interaction between bottom-up perceptions and top-down processing and also modulates the level of certainty of top-down information. The authors suggested that cholinergic deficits lead to over-processing of top-down information leading to visual hallucinations [24]. An imbalance between serotonin and acetylcholine (ACh) has also been implicated in visual hallucinations [25].

**Fig. 1:**
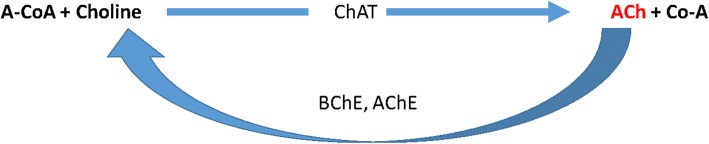
Acetylcholine (ACh) is synthesised by choline acetyltransferase (ChAT) and broken down by acetylcholinesterase (AChE) at low concentrations and butyrylcholinesterase (BChE) at higher concentrations

We set out to determine whether visual hallucinations in AD result from Lewy body pathology or chronic hypoperfusion affecting the visual processing areas in the occipital cortex. Given that acetylcholinesterase inhibitors reduce visual hallucinations (at least to some extent) and the suggestion of a direct link between cholinergic input and vessel profusion, we also assessed cholinergic input, by measuring the 3 enzymes involved in the production and breakdown of ACh (see Fig. 1). We previously demonstrated that the ratio of the hypoxia-sensitive myelin-associated glycoprotein (MAG) to the hypoxia-resistant proteolipid oprotein-1 (PLP) is a robust measure of ante-mortem perfusion in relation to metabolic demand [26, 27]. Both myelin proteins are very stable under post-mortem conditions [26] and have half-lives of several months. A decline in the MAG:PLP1 ratio in post-mortem brain tissue reflects a reduction in ante-mortem oxygenation of the tissue over a period of several months prior to death. von Willebrand factor is a marker of microvessel (capillary) density, and VEGF is upregulated in hypoperfused tissue [18, 26–28]. We previously showed that VEGF and capillary density are reduced in the primary visual cortex in DLB [18].

**Fig. 2:**
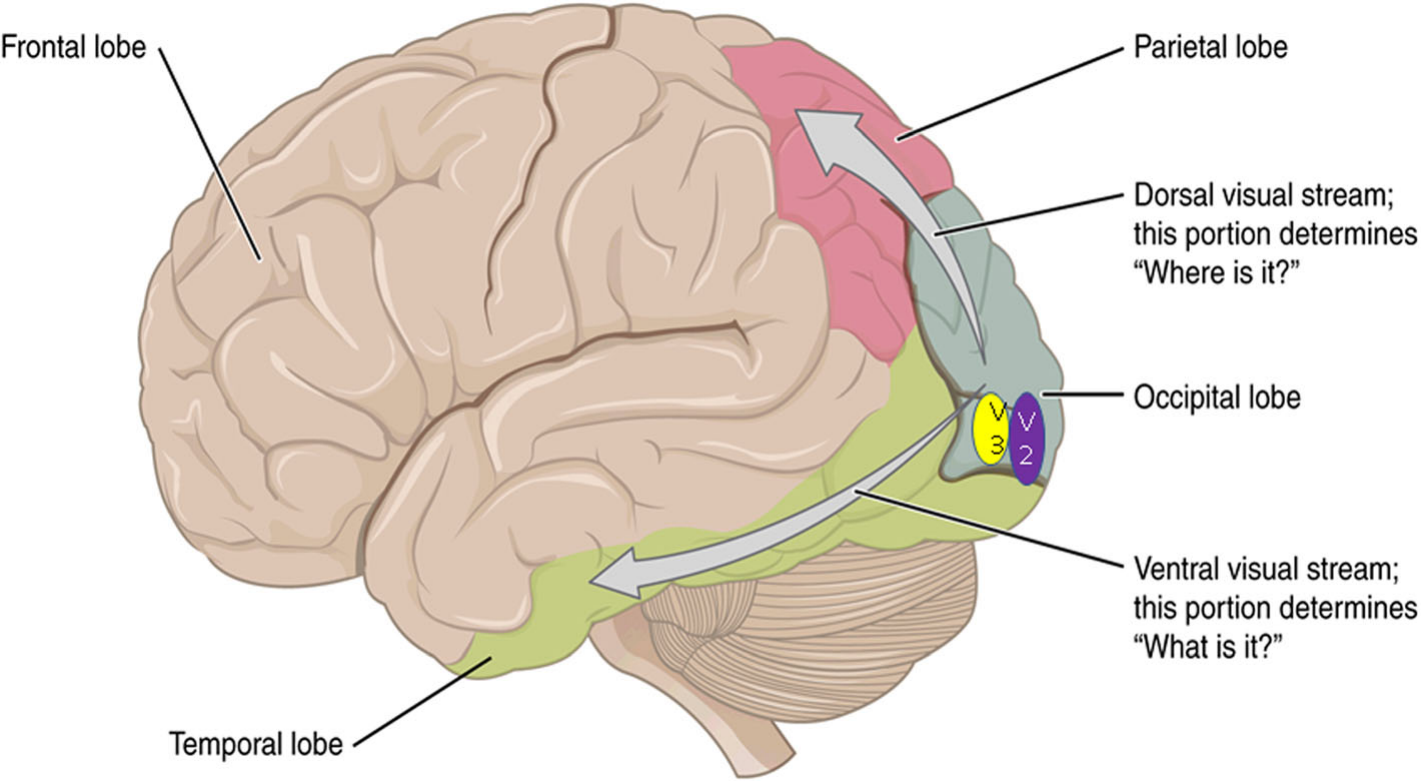
The visual processing pathways in the brain. Ventral visual area V2 is shown in purple (BA18) and ventral visual area V3 is shown in yellow (BA19). Figure from the OpenStax Anatomy & physiology textbook 2014 CCBY 3.0, available from https://cnx.org/contents/FPtK1zmh@12.16:KcreJ7oj@9/Central-Processing

In view of the evidence of reduced perfusion in the occipital lobes in patients with visual hallucinations, and the theoretical implication of visual processing areas in the occipital cortex, we decided to focus on V2 (BA18) and V3 (BA19). After V1 (BA17), the visual processing pathway splits into ventral and dorsal pathways (see Fig. 2). In the present study, we have assessed the ventral pathway, as this is postulated to be concerned with “what” is seen rather than the “where” that is processed via the dorsal pathway. There is evidence to suggest that the ventral pathway is more likely to be involved in visual hallucinations in DLB [23, 29].

We hypothesised that the occipital cortex from AD patients with visual hallucinations would show evidence of reduced perfusion as measured by the MAG:PLP1 ratio in V2 and V3 compared to controls and to AD patients without visual hallucinations.

## Methods

The study used brain tissue and clinical data obtained from the South West Dementia Brain Bank (SWDBB), University of Bristol, with local Research Ethics Committee approval (REC reference 18/SW/0029). Comprehensive clinical summaries are recorded in the SWDBB database for all brain donors. The summaries are generated from the patient medical notes and from any additional information collected as part of the Brains for Dementia Research Programme. The summaries were searched using the following terms: psychosis, hallucinating, hallucinations, quetiapine, risperidone and visual. This allowed us to identify 24 individuals with neuropathologically confirmed AD who had definitely experienced visual hallucinations and 33 individuals with AD who had definitely not experienced visual hallucinations during their illness. We also selected 23 individuals with neuropathologically confirmed dementia with Lewy bodies, 2 of whom had not and 21 who had experienced visual hallucinations, and 36 controls without dementia. We excluded individuals who had had major vascular abnormalities or vascular dementia as a primary diagnosis. Psychiatric, neurological and treatment histories for the individuals in this cohort are summarised in Additional file 1: Table S3. The cohorts were matched for age, gender and post-mortem interval. Braak tangle stage and severity of arteriolosclerotic small vessel disease and cerebral amyloid angiopathy had been assessed as described [26, 30].

Dementia was assessed clinically using DSM-IV criteria [31], and DLB was diagnosed according to consensus neuropathological criteria [4]. The right half of each brain had been fixed in formalin for 3 weeks prior to the detailed neuropathological examination, and the left half sliced and subdivided into multiple samples which were frozen at − 80 °C. Neuropathological diagnoses of AD were made according to NIA-AA criteria [32]. Control individuals had no history of cognitive problems or psychosis during their lifetime and had no major neuropathological abnormalities on post-mortem examination other than mild neurofibrillary tangle pathology (Braak stage ≤ 3) or scattered diffuse Aβ plaques (see Table 1 for a summary of the characteristics of the cohorts). The cases selected for the dementia with Lewy bodies group had only “low AD neuropathologic change” which according to NIA-AA criteria [32] would not have accounted for dementia.

**Table 1:** Characteristics of the study cohort. Small vessel disease and cerebral amyloid angiopathy scoring were performed on a 4-point scale [26, 30]. Although the cohorts were matched for age, gender and post-mortem interval, there was weak evidence of a between-group difference in age at death. Analyses were therefore carried out both with and without age as a covariate

	AD with visual hallucinations, *n* = 23		AD without visual hallucinations, *n* = 19		Dementia with Lewy bodies, *n* = 19		Controls, *n* = 36		Statistical findings	*p* with age as covariate
	Mean	SD	Mean	SD	Mean	SD	Mean	SD		
Age (years)	76.7	9.0	79.3	9.0	81.0	7.0	82.1	6.3	ANOVA *p* = 0.065	
Post-mortem interval (h)	36.7	21.1	41.8	16.3	33.3	18.1	36.9	15.6	ANOVA *p* = 0.533	
Age at onset of dementia (years)	66.9	10.4	66.4	10.1	73.0	6.9	N/A	N/A	Kruskal-Wallis *p* = 0.164, *χ*^2^ = 3.611	
Duration of dementia (years)	9.7	4.2	11.1	3.4	7.6	4.6	N/A	N/A	ANOVA *p* = 0.04	0.0340
Mean Braak stage	5.5	0.5	5.3	0.9	3.1	1.5	2.0	0.8	*χ*^2^ = 109.9, *p* < 0.001	
Cerebral amyloid angiopathy score										
0	3		3		9		24		*χ*^2^ = 31.37, *p* < 0.001	
1	7		3		3		3			
2	7		8		2		3			
3	6		4		5		3			
Occipital small vessel disease score										
0	2		2		3		8		*χ*^2^ = 8.07, *p* = 0.527	
1	11		6		5		10			
2	6		3		1		6			
3	0		2		1		1			

### Homogenate preparation

Fresh frozen tissue was dissected from ventral BA18 and BA19 from the left occipital lobe. To produce the SDS homogenates used for most of the assays in this project, 200 mg of tissue was homogenised in 1 ml of chilled 1% SDS lysis buffer in a Precellys tissue homogeniser (2 × 15 s at 6000*g*) with 6–10 zirconia beads in a 2-ml homogenate tube. The homogenates were then centrifuged for 15 min at 13,000*g* at 4 °C. The supernatant was aliquoted into non-binding 96-well storage plates (Thermo Scientific) and frozen at − 80 °C until required.

Choline acetyltransferase (ChAT) homogenates were prepared using a method adapted from that of Peng et al. [33]. Fifty to 100 mg of fresh frozen tissue was placed in a 2-ml homogenate tube with 6–10 zirconia beads. The volume of all buffers in microlitres added was calculated as 15× the tissue weight in milligrams. Buffer A was 50 mM potassium phosphate, 2 mM EDTA, pH 7.4. Buffer B was 50 mM potassium phosphate, 2 mM EDTA, 500 mM NaCl, pH 7.4. Buffer C was 50 mM potassium phosphate, 2 mM EDTA, 0.6% triton X-100, pH 7.4. After each buffer was added, the tissue was homogenised for 2 × 15 s at 6000*g* in a Precellys tissue homogeniser and then spun for 30 min at 28,000×*g* at 4 °C. The homogenisation was started with buffer A. The supernatant was removed, and the tissue re-homogenised and then re-centrifuged in the subsequent buffers. In this way, the fractions extracted were soluble ChAT (buffer A), ionic membrane-bound ChAT (buffer B) and membrane-bound ChAT (buffer C). The total ChAT homogenates used in the assays described below were prepared by adding equal volumes of each supernatant, e.g. 200 μl supernatant A plus 200 μl supernatant B plus 200 μl supernatant C. As no protease inhibitors were used in the sample preparation, the whole operation was carried out with the samples either on ice or at 4 °C. The samples were aliquoted out (100 μl per tube) and frozen at − 80 °C until required. The reason for omitting protease inhibitors cocktail in the buffers is that one or several of these inhibitors also irreversibly inhibit the cholinergic enzymes (personal observations by TDS) and thereby would interfere with the downstream enzyme activity assessments.

The soluble and insoluble extracts used for the α-synuclein assays were prepared in a third buffer. Up to 200 mg of tissue was homogenised in 1 ml of chilled TBS extraction buffer with 1% NP-40 (pH 7.4) in a Precellys tissue homogeniser (2 × 15 s at 6000*g*) with 6–10 zirconia beads in a 2-ml homogenate tube. The homogenates were then centrifuged for 15 min at 13,000*g* at 4 °C. The supernatants were removed and aliquoted into non-binding 96-well storage plates (Thermo Scientific) and frozen at − 80 °C until required. The pellet was re-suspended in 400 μl of guandicine HCl buffer (3 parts guanidine HCl to 1 part 50 mM Tris, pH 8), homogenised again in a Precellys tissue homogeniser (2 × 15 s at 6000*g*) and then spun for 15 min at 13,000*g* at 4 °C. The supernatant was removed, aliquoted onto non-binding 96 well storage plates (Thermo Scientific) and frozen at − 80 °C until required. Total protein was measured for all samples with a Coomassie Protein Plus kit (Thermo Scientific).

### Vascular endothelial growth factor ELISA

VEGF was measured using a commercial kit (R&D Systems Duoset DY293B) adapted for use in a 384-well Nunc MaxiSorp plate. Seventy-five microlitres of mouse anti-human VEGF capture antibody, diluted 1:120 in PBS, was added to each well. The plate was then sealed and incubated overnight at room temperature. Following 4 × 3-min washes with 0.05% PBS/Tween per well, the plate was blocked by adding 100 μl 1% BSA/PBS to each well and was incubated for 1 h at 26 °C with agitation. After a further 4 × 3 min with 110 μl 0.05% PBS/Tween per well, the samples, standards and blanks were added in triplicate. The samples were diluted 1:10 in 1% BSA/PBS and 50 μl added to each sample well. Blanks were 50 μl of 1% BSA/PBS. Standards were prepared in line with the manufacturer’s instructions, and 50 μl was added to each well. The plate was incubated for 2 h at 26 °C with agitation. After a further 4 washes, 75 μl of biotinylated goat anti-human VEGF detection antibody (diluted 1:60 in 1% BSA/PBS) was added to each well, and the plate then incubated for 2 h at 26 °C plus agitation. After a further 4 washes, 75 μl of streptavidin HRP diluted 1:40 in PBS/0.05% Tween was added to each well and the plate incubated for 20 min in the dark at room temperature. After a final 4 washes, 50 μl of the substrate was added to each well, and 25 μl of STOP solution was added after 20 min. Absorbance was read at 450 nm. The intraclass correlation coefficient for this assay was 0.89, indicating excellent consistency.

### Myelin-associated glycoprotein ELISA

MAG was measured by indirect ELISA which was developed in-house [26, 27]. Autoclaved PBS was used throughout this assay. Samples were diluted 1:10 in autoclaved PBS and loaded in duplicate onto a 96-well Nunc MaxiSorp ELISA plate. Blanks consisted of 100 μl of PBS and standards (Abcam ab89780) ranged from 547.6 to 8.56 ng/ml. The plate was incubated for 2 h at 26 °C with agitation. After 5 washes in PBS/0.05% Tween, the plate was blocked by adding 300 μl of 1%BSA/PBS per well and incubated for 1 h at 26 °C with agitation. After a further 5 washes, 100 μl of mouse monoclonal anti-MAG antibody (Abcam ab89780) diluted 1:1000 in PBS was added to each well. The plate was incubated for 2 h at 26 °C with agitation. After a further 5 washes, 100 μl of biotinylated anti-mouse antibody (Vector, diluted 1:4000 in PBS) was added to each well, and the plate incubated for 20 min at 26 °C with agitation. After a further 5 washes, 100 μl of streptavidin-HRP diluted 1:500 in PBS/0.01% Tween was added to each well and the plate incubated for 20 min at 26 °C with agitation. After a final 5 washes, 100 μl of the substrate was added to each well and 50 μl of STOP solution added after 10 min. Absorbance was read at 450 nm. The intraclass correlation coefficient for this assay was 0.60, indicating good consistency. All samples were assayed in duplicate on each plate and on two different plates.

### PLP ELISA

PLP was assayed using a commercial sandwich ELISA kit (Cloud-Clone Corporation), as per the manufacturer’s instructions. Samples were diluted 1:10 for this assay. Three samples were carried over on each plate to check that the results were consistent.

### von Willebrand factor (vWF) dot blot

Samples were initially centrifuged again for 20 min at 15,000 rpm to remove any particulate matter then diluted 1:400 in TBS. The standard was 7 serial dilutions of a reference homogenate, starting with a dilution of 1:100 in TBS. Prior to assembling the dot blot manifold, the membrane was soaked in TBS for at least 10 min. One hundred microlitres of each sample was loaded onto the membrane after it had been placed in the dot blot vacuum manifold and was incubated for 75 min. The manifold was then disassembled and the membrane washed for 3 × 10 min in TTBS prior to blocking in 5% milk/TBS for 1 h at room temperature with agitation. After a further 3 × 10-min washes in TTBS, the membrane was incubated overnight at 4 °C with agitation, in rabbit polyclonal anti-vWF antibody (Dako A0082) diluted 1:3000 in 5% milk/TTBS. Following 3 × 30-min washes, the membrane was incubated in HRP conjugated goat anti-rabbit antibody (Vector) diluted 1:5000 in 5% milk/TTBS for 1 h at room temperature with agitation. After a further 3 × 30-min washes, 6 ml of the substrate was added to each membrane, and after 4 min, it was imaged in a BioRad imager (chemiluminescence protocol). All samples were assayed in duplicate on each membrane and on two different membranes. The intraclass correlation coefficient for this assay was 0.69, indicating good consistency.

### Alpha-synuclein ELISA

Alpha-synuclein was measured in soluble and insoluble extracts by ELISA developed in-house, as described previously, adapted for use in a 384-well Nunc MaxiSorp plate [34, 35]. The mouse monoclonal capture antibody (BD Biosciences) was diluted 1:1000 in PBS and 75 μl added to each well and left overnight at room temperature. After 5 washes in PBS/0.05% Tween, the plate was blocked by adding 100 μl of 1% BSA/PBS to each well and incubating the plate at room temperature for 70 min with agitation. After a further 5 washes, the samples, 7 serial twofold dilutions of recombinant protein standard (R peptide S-100-1) and blanks were loaded, 50 μl per well in duplicate. The concentration of the standards ranged from 62.5 to 0.98 ng/ml. The samples were diluted 1:400 in PBS, and the blanks consisted of 50 μl PBS. The plate was incubated at room temperature for 90 min plus agitation. After a further 5 washes, 70 μl of biotinylated polyclonal antibody (R&D systems BAF1338), diluted 1:600 in 1% BSA/PBS, was added to each well and the plate incubated for 90 min at room temperature with agitation. After a further 5 washes, 75 μl of streptavidin-HRP diluted 1:200 in PBS/0.05% Tween was added to each well and the plate incubated for 20 min in the dark at room temperature with agitation. After a final 5 washes, 50 μl of the substrate was added to each well, and 25 μl of STOP solution was added after 20 min. Absorbance was read at 450 nm.

### Measurement of ChAT, AChE and BChE

ChAT activity was assayed using an assay designed by Dr. Darreh-Shori’s group at the Karolinska Institutet, as previously described in detail [36]. The assay was performed on 384-well plates. The wells were coated overnight at 4 °C with 100 μl of monoclonal anti-ChAT (R&D systems MAB3447) diluted 1:250 in 1× coating buffer per well except for wells which would later be used for the choline standards. After 5 washes with TBS, the samples (both native and denatured), standards and blanks were loaded. Samples were denatured by incubation at 99 °C for 3 × 8 min 30 s in a PCR machine. Ten microlitres of each sample was loaded in triplicate. Blanks consisted of dilution buffer alone. The reference standard for ChAT concentration was a pooled brain homogenate which had been prepared with 10× vol of each buffer. There were 7 × 2-fold dilutions of the pooled homogenate, starting with a dilution of 1:2. The choline standards for ChAT activity consisted of 8 × 2-fold dilutions of choline, with choline standard C1 = 50 μM. Fifty microlitres of each choline standard was loaded in triplicate. Forty microlitres of cocktail A was added to each sample/pooled homogenate/blank well. cocktail A was prepared as 4× of final concentrations and contained 10 μM choline chloride, 50 μM acetyl-CoA, 7 mM acetyl phosphate and 1 unit/ml phosphoacetyltransferase (to continuously regenerate acetyl-CoA from –CoA generated by ChAT) in TBS buffer.

The plate was then incubated for 1 h at 38.5 °C with gentle agitation. After this, 25 μl of cocktail B was added to each well including those containing the choline standards, and the plate was immediately read at 500 nm, at 1 min interval for 1 h, using the kinetic mode of a Tecan M1000 spectrophotometer. The cocktail B contained 2.1 mM phenol, 1 mM 4-aminoantipyrine, 0.31 units/ml choline oxidase and 1/15,000 streptavidin HRP in 10 mM PBS, pH 7.6.

The plate was then sealed and left overnight at 4 °C. The next day, the plate was read again at 500 nm prior to 3 × 5-min washes in TBS/0.05% Tween. One hundred microlitres of TBS/0.05% Tween/5% BSA/0.1% sodium azide was loaded into each well, and the plate was incubated for 30 min at 38 °C with gentle agitation. After a further 3 washes, 100 μl of polyclonal anti-ChAT antibody (Abnova PAB14536) diluted 1:3500 in TBS/0.05% Tween/1% BSA/0.01% sodium azide was loaded per well, and the plate was incubated for 1 h at 38 °C with gentle agitation. After a further 3 washes, 75 μl of polyclonal swine anti-rabbit AP diluted 1:1750 in TBS/0.05% Tween/1% BSA/0.01% sodium azide was loaded per well, and the plate incubated for 30 min at 38 °C with agitation. After a further 4 washes in TBS/0.05% Tween and one wash in diethanolamine buffer (0.022% DEA, 50 mM MgCl_2_, pH 9.8), 75 μl of AP substrate (para-nitrophenol, 1 mg/ml) was loaded into each well, and the plate was read at 405 nm, with a reference absorbance of 650 nm. The absorbance was read immediately, at 1 h, 2 h and the next morning.

AChE and BChE assays were performed using a modified version of Ellman’s colorimetric assay adapted to a high-throughput assay as described previously [37, 38]. Briefly, 384-well Nunc MaxiSorp plates (Merck) were coated O/N at 4 °C with 75 μl of 1:1000 HR2 (anti-AChE monoclonal antibody; Thermo Fisher) and 1:2000 diluted 3E8 *Ab17246* in carbonate buffer pH 9.5, (anti-BChE monoclonal antibody; Abcam) for AChE and BChE assays, respectively. The next day, the plates were emptied and washed three times with TBS (50 mM Tris-Cl, pH 7.5 and 150 mM NaCl) buffer followed by blocking with carbonate buffer pH 9.5, containing 5% BSA for 1 h at room temperature (RT). Afterwards, the plates were washed three times with TBS buffer containing 0.05% Tween-20 (TBS-T) and 25 μl of standards, and samples from ventral BA18 and the left occipital lobe BA19 were added in triplicate to the assigned wells. For standards, the human recombinant-AChE (S1-S8 with 2 times serial dilution starting from 200 ng/ml; Sigma-Aldrich, C1682) and human-BChE (S1-S8 with 2 times serial dilution starting from 34.3 ng/ml; Sigma-Aldrich, C9971) were used. Next, 50 μl of master mix prepared in Na/K phosphate buffer, containing final concentrations of 0.5 mM acetylthiocholine iodide (ATC) or 5 mM butyrylthiocholine iodide (BTC), 0.4 mM 5,5′-dithiobis (2-nitrobenzoic acid) (DTNB) and 0.1 mM ethopropazine (BChE inhibitor) or 1 μM BW284c51 (AChE inhibitor; Sigma-Aldrich, A9013), were added to the AChE and BChE assay plate wells. The plates were read at 412 nm for 30 min with 2-min interval cycle using a microplate spectrophotometer reader (Infinite M1000, Tecan) to measure AChE and BChE activity (ΔOD/min).

For AChE and BChE protein concentration estimation, an ELISA-like functional assay was used. Briefly, the plates were sealed following activity measurement and incubated for 2–3 h at RT. After incubation, the plates were washed two times with TBS-T buffer and read again at 412 nm for 60 min with 5-min interval cycle after adding master mix containing final concentrations of 0.5 mM ATC or 5 mM BTC and 0.4 mM DTNB. The protein levels of AChE and BChE in the samples were then determined from the linear part of the standard curves as described before [39].

### Formalin-fixed tissue preparation

The right cerebral hemisphere of each brain had been fixed for 3–4 weeks in formalin prior to being dissected into blocks and embedded in wax. The paraffin sections of the occipital cortex containing both BA18 and BA19 were cut at 7 μM thickness. Immunolabelling for alpha-synuclein was performed in an automated immunostainer. The mouse monoclonal anti α-synuclein antibody (Vector VP-A106) was diluted 1:800 and applied for 1 h prior to DAB development of the slides.

The field fraction immunopositive for each antigen was assessed by examining up to 15 random fields of BA18 and BA19 under a × 20 objective. The software package Image-Pro Plus (Media Cybernetics, MD, USA) was used to select the fields at random in the pre-defined area and for image capture. For a small number of slides, the area of BA18/19 included in the section was too small to accommodate 15 non-overlapping fields, but at least 10 were captured in all cases.

### Statistical analysis

All ELISAs were adjusted for total protein content. This was done using the following formula:

$$ \mathrm{Adjusted}\ \mathrm{result}=\frac{\mathrm{sample}\ \mathrm{target}\ \mathrm{protein}\ \mathrm{concentration}}{\mathrm{sample}\ \mathrm{total}\ \mathrm{protein}\ \mathrm{concentration}} $$

All data were analysed using parametric statistical tests such as ANOVA wherever possible. If a variable was not normally distributed and it was not possible to achieve a normal distribution by transformation, non-parametric tests were used such as the Kruskal-Wallis test. The primary outcome measure was whether the MAG:PLP1 ratio varied between the groups. For field fraction analysis, each field on each slide was given equal weighting, and mean values were compared by ANOVA, with age, gender and post-mortem interval as covariates. For a priori analyses, any demographic variable which varied between groups (e.g. age, post-mortem interval) was included as a covariate. Where Dunn’s test was used, Bonferroni correction was applied. A threshold *p* value of 0.05 was used throughout.

We estimated, using the information from a previous study [18], that we would have 89% power to detect a between-group difference in the MAG:PLP1 ratio of 0.07.

## Results

Tissue was obtained from a total of 97 individuals for both BA18 (visual processing area 2) and BA19 (visual processing area V3): 23 with AD and a history of visual hallucinations, 19 individuals who had Alzheimer’s disease who had not suffered from visual hallucination, 19 individuals who had dementia with Lewy bodies (either with or without visual hallucinations) and 36 controls. The groups were matched for age, gender and post-mortem interval, although the AD + visual hallucinations group was marginally younger than the other groups (*p* = 0.065). As would be expected, the AD groups had a higher Braak stage than the other groups, and there was a between-group difference in cerebral amyloid angiopathy score, with both AD groups scoring higher [30]. Although the initial ANOVA was suggestive of a difference between the groups in disease duration, there was little evidence of a difference in the subsequent regression.

As shown in Additional file 1: Table S2, the clinical diagnosis during life frequently differed from the neuropathological diagnosis. This was more likely for the AD with visual hallucinations and dementia with Lewy body groups. The rate of change of diagnosis was similar to other post-mortem series [40, 41].

Because of the marginal difference in age between the groups, parametric analyses were carried out both with and without age as a covariate to exclude the confounding effect of a difference in age as the explanation for any between-group differences observed.

**Fig. 3:**
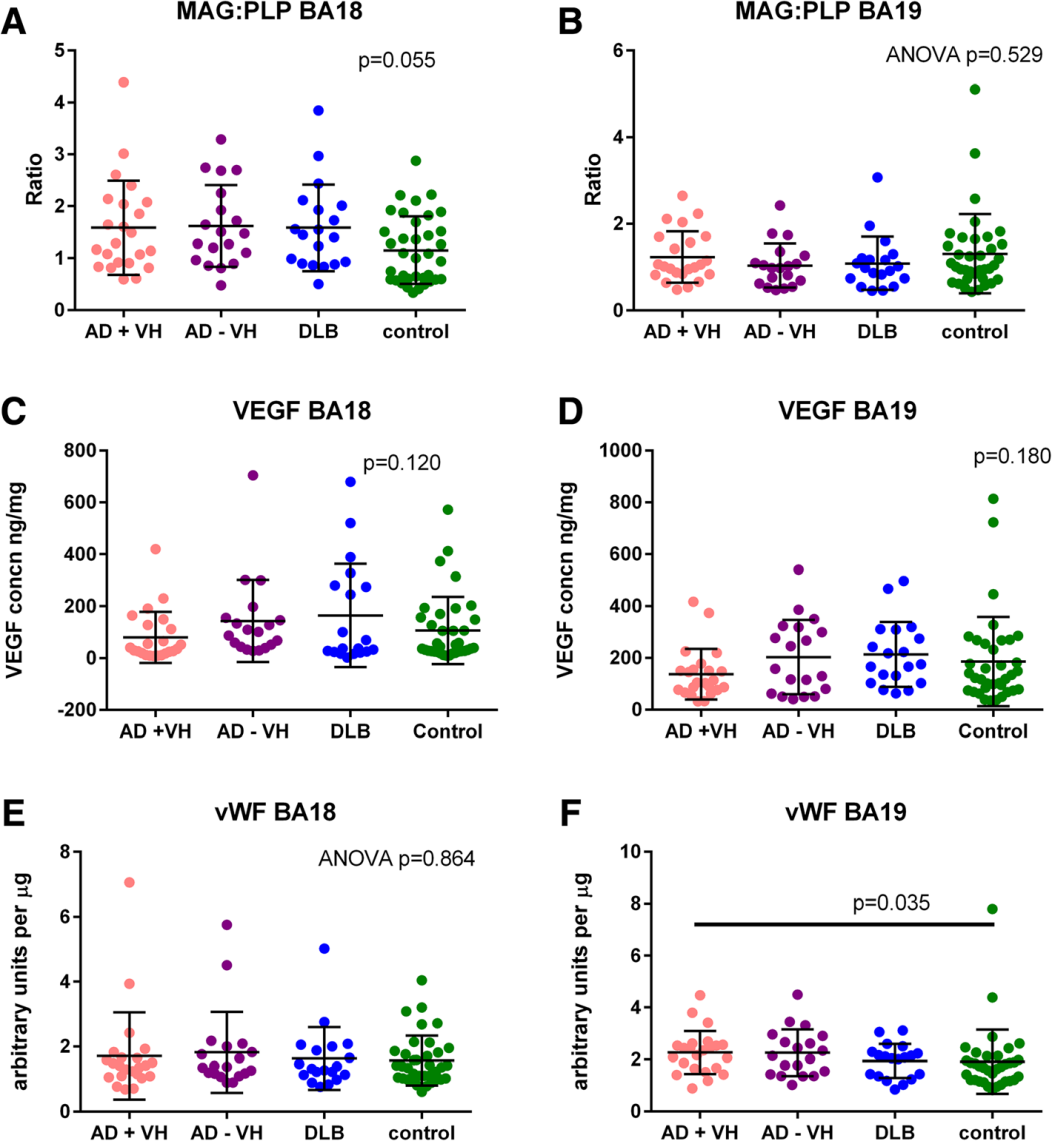
There was no between-group difference in MAG:PLP1 in BA19 (**b**), but all three dementia groups seemed to have increased MAG:PLP1 in BA18 (**a**). There was no convincing between-group difference in VEGF in either BA 18 (**c**) or BA19 (**d**) or in vWF in BA18 (**e**). There was some evidence that vWF was increased in the AD + VH group in BA19 (**f**)

As can be seen in Table 2 and Fig. 3, there was no strong evidence of a between-group difference in VEGF, MAG, PLP1 or vWF. There was no evidence of a between-group difference in MAG:PLP1 in BA19, but unexpectedly, MAG:PLP1 seemed to be increased in all three dementia groups in BA18. There was only statistical evidence of a between-group difference between the two AD groups (*p* = 0.029). As expected from previous studies, MAG:PLP1 correlated negatively with VEGF (see Additional file 1: Figure S1) [18].

**Table 2:** Results of the hypoperfusion-related ELISAs carried out in this study

	AD with visual hallucinations, *n* = 23		AD without visual hallucinations, *n* = 19		Dementia with Lewy bodies, *n* = 19		Controls, *n* = 36		Statistical findings	Age as covariate	Statistics with controls with no Psych Hx or CVAs
	Mean	SD	Mean	SD	Mean	SD	Mean	SD			
VEGF in BA18 (ng/mg)	79.987	98.734	143.059	158.859	164.705	198.983	106.731	129.774	Kwallis, *χ*^2^ = 5.872, *p* = 0.120		Kwallis, *χ*^2^ = 5.463, *p* = 0.140
VEGF in BA19 (ng/mg)	136.886	97.292	203.243	143.618	213.453	125.463	186.541	172.574	Kwallis, *χ*^2^ = 4.894, *p* = 0.180		Kwallis, *χ*^2^ = 6.836, *p* = 0.077
MAG in BA18 (μg/mg)	50.359	47.968	48.091	8.147	45.326	15.884	43.788	7.183	Kwallis, *χ*^2^ = 7.610, *p* = 0.055		
MAG in BA19 (μg/mg)	72.119	20.574	66.889	17.848	64.974	9.888	72.966	22.934	ANOVA *p* = 0.529	*p* = 0.486	
MAG:PLP1 in BA18	1.587	0.905	1.62	0.784	1.585	0.835	1.154	0.649	ANOVA *p* = 0.022	*p* = 0.034	Kwallis, *χ*^2^ = 1.396, *p* = 0.701
MAG:PLP1 in BA19	1.233	0.59	1.038	0.507	1.089	0.614	1.31	0.913	ANOVA *p* = 0.534	*p* = 0.541	Kwallis, *χ*^2^ = 2.704, *p* = 0.440
PLP1 in BA18 (ng/μg)	40.821	40.649	38.583	24.223	36.472	21.212	50.426	26.188	Kwallis, *χ*^2^ = 6.357, *p* = 0.096		
PLP1 in BA19 (ng/μg)	69.523	34.204	75.993	36.68	74.969	35.674	74.161	42.655	ANOVA *p* = 0.920	*p* = 0.915	
von Willebrand factor in BA18 (arbitrary units/μg)	1.713	1.345	1.824	1.246	1.636	0.968	1.568	0.767	ANOVA *p* = 0.864	*p* = 0.842	Kwallis, *χ*^2^ = 4.580, *p* = 0.205
von Willebrand factor in BA19 (arbitrary units/μg)	2.267	0.832	2.26	0.896	1.94	0.653	1.909	1.234	Kwallis, *χ*^2^ = 8.223, *p* = 0.042		Kwallis, *χ*^2^ = 5.916, *p* = 0.116

**Fig. 4:**
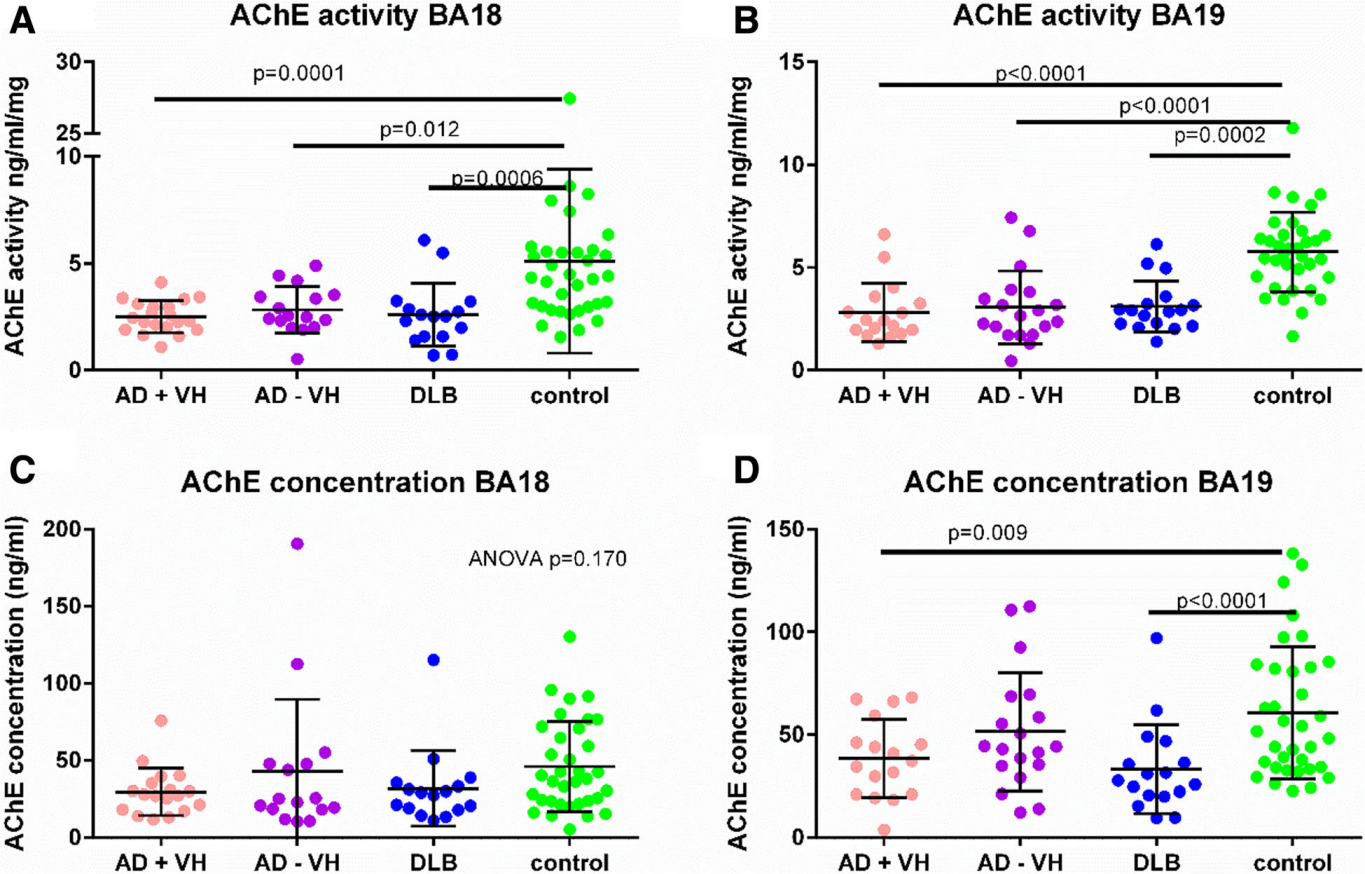
Acetylcholine breakdown enzymes. AChE activity was reduced in all three dementia groups in BA18 (**a**) and BA19 (**b**). AChE concentration was decreased in the AD with VH and DLB groups in BA19 (**d**) but was unaltered in BA18 (**c**). VH, visual hallucinations

There was no evidence of a between-group difference in ChAT activity (Table 3, Additional file 1: Figure S3). AChE activity was reduced in all three dementia groups in both BA18 and BA19, as shown in Fig. 4. AChE concentration was unchanged in BA18 but was decreased in the AD with visual hallucinations and DLB groups in BA19. There was no evidence of a between-group difference in either BChE activity or concentration (see Additional file 1: Figure S4). ChAT activity, AChE and BChE were relatively stable with disease duration (Additional file 1: Figure S6). There was no evidence of a change in the ratio of cholinergic breakdown to production enzymes (known as the cholinergic index), as shown in Additional file 1: Figure S5.

Removing controls with a history of mental health problems, stroke or TIAs reduced the number of controls to 16. The findings in relation to AChE, BChE and ChAT were unchanged when these controls were excluded. The evidence for a between-group difference in MAG to PLP ratio in BA18 in the opposite direction to that predicted weakened considerably, but this did not affect our overall conclusion that there was no strong evidence of hypoperfusion in the occipital lobes of those with AD and visual hallucinations.

**Fig. 5:**
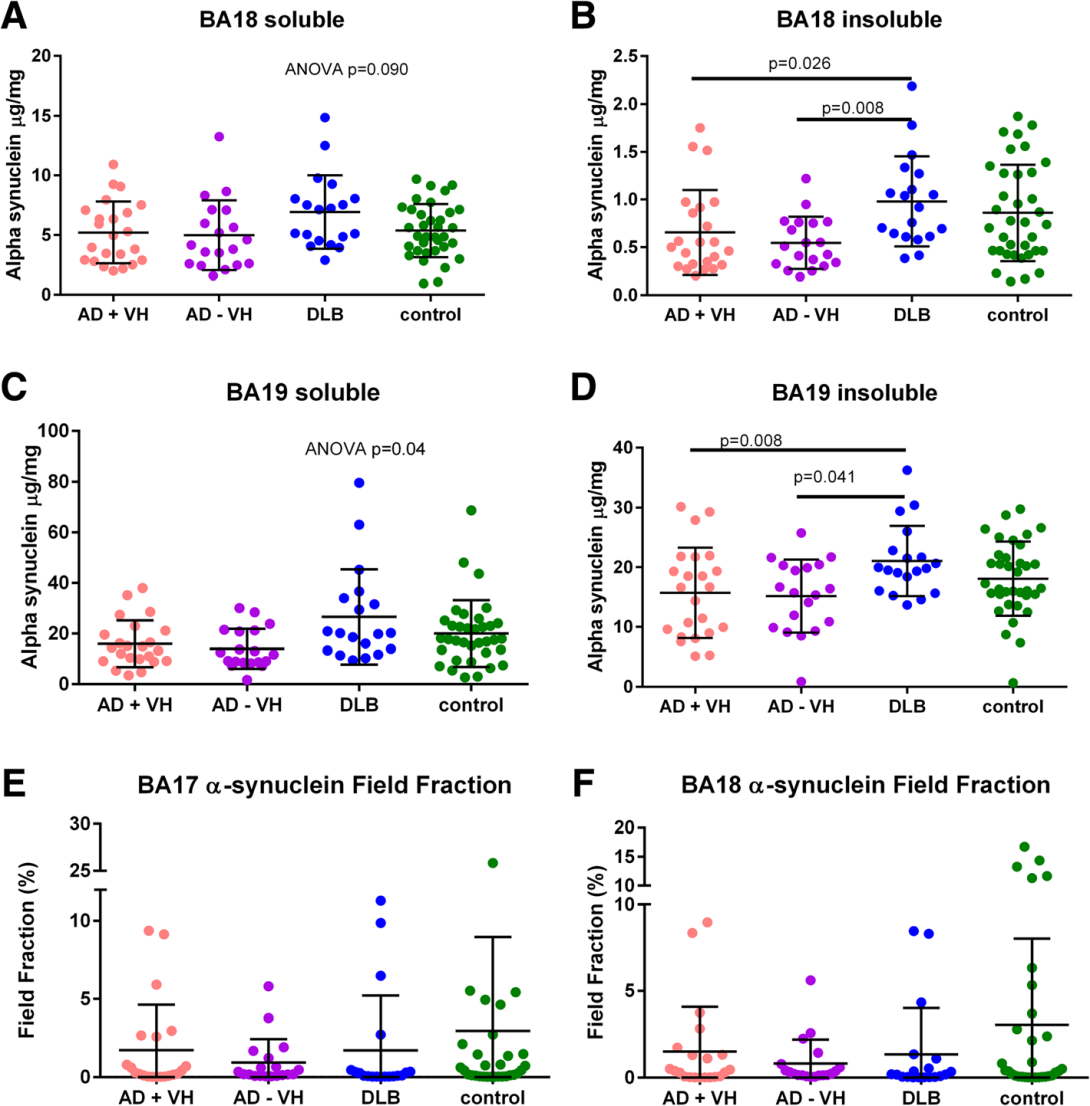
**a**–**f** Results of α-synuclein ELISAs and field fraction analysis. There was no strong evidence of a between-group difference in soluble α-synuclein. There was increased insoluble α-synuclein in the DLB group compared to both AD groups, but the DLB group did not differ statistically from controls. VH, visual hallucinations

Field fraction analysis yielded no evidence of a between-group difference in immunolabelled α-synuclein, but most of the values were very small (see Fig. 5 and Additional file 1: Table S4). Measurement of soluble and insoluble fractions by ELISA showed that insoluble α-synuclein was increased in the DLB group in BA18 and 19 compared to both AD groups, as would be expected. In both brain areas, although the initial ANOVA was suggestive of a between-group difference in soluble α-synuclein, this was not shown in the subsequent regression.

## Discussion

In this relatively large post-mortem study of AD and DLB, we did not find any strong evidence of occipital hypoperfusion or α-synuclein accumulation in the tissue from AD patients with visual hallucinations. There was evidence of derangement of the cholinergic equilibrium in AD and DLB: a reduction in acetylcholinesterase activity most marked in AD with visual hallucinations, but no change in the ChAT activity.

**Table 3:** Results of the cholinergic system ELISAs carried out in this study

	AD with visual hallucinations, *n* = 23		AD without visual hallucinations, *n* = 19		Dementia with Lewy bodies, *n* = 19		Controls, *n* = 36		Statistical findings	*p* with age as covariate	Statistics with controls with no Psych Hx or CVAs
	Mean	SD	Mean	SD	Mean	SD	Mean	SD			
AChE concentration in BA18 (ng/ml)	28.823	14.693	42.551	47.023	31.811	24.617	45.924	29.15	ANOVA *p* = 0.170	*p* = 0.169	*p* = 0.174
AChE activity in BA18 (nmol/min/mg)	2.504	0.746	2.833	1.102	2.597	1.47	5.09	4.297	Kruskal-Wallis, *p* = 0.0001, *χ*^2^ = 24.415		Kruskal-Wallis, *p* = 0.012, *χ*^2^ = 10.984
AChE concentration in BA19 (ng/ml)	38.412	19.008	51.382	28.809	33.193	21.513	60.627	32.241	*p* = 0.002	p = 0.002	*p* = 0.011
AChE activity in BA19 (nmol/min/mg)	2.825	1.429	3.064	1.774	3.11	1.248	5.752	1.948	Kruskal-Wallis, *p* = 0.0001, *χ*^2^ = 35.2		Kruskal-Wallis, *p* = 0.001, *χ*^2^ = 16.506
BChE concentration in BA18 (ng/ml)	4.123	4.269	3.944	4.054	2.745	3.181	3.397	3.417	Kruskal-Wallis, *p* = 0.819, *χ*^2^ = 0.927		*p* = 0.739
BChE activity in BA18 (nmol/min/mg)	3.482	1.052	3.623	1.844	3.13	1.629	3.757	2.64	ANOVA *p* = 0.652	*p* = 0.603	*p* = 0.554
BChE concentration in BA19 (ng/ml)	7.798	7.298	5.481	2.786	6.34	7.804	6.783	5.102	Kruskal-Wallis, *p* = 0.779, *χ*^2^ = 1.097		Kruskal-Wallis, *p* = 0.573, *χ*^2^ = 1.995
BChE activity in BA19 (nmol/min/mg)	3.842	2.402	3.479	1.618	4.121	2.136	3.932	1.806	Kruskal-Wallis, *p* = 0.749, *χ*^2^ = 1.213		Kruskal-Wallis, *p* = 0.859, *χ*^2^ = 0.762
ChAT activity in BA18 (pmol/min/mg)	1.092	0.791	1.333	0.896	1.1	0.545	1.35	1.11	ANOVA *p* = 0.709	*p* = 0.829	*p* = 0.150
ChAT activity in BA19 (pmol/min/mg)	5.574	10.948	2.174	3.443	2.433	2.533	1.28	1.021	Kruskal-Wallis, *p* = 0.646, *χ*^2^ = 1.658		Kruskal-Wallis, *p* = 0.723, *χ*^2^ = 1.326

It is well known that cholinergic innervation is lost in AD [42], although the occipital lobe seems to be relatively spared compared to the other brain regions [43]. It was originally thought that this loss of cholinergic neurons occurred earlier in AD than in DLB, but more recent work suggests the converse [44]. The temporal cortex may be affected earlier than the other brain areas in AD. Tiraboschi et al. [44] found cholinergic loss to be greatest in the superior temporal cortex in mild AD, less in the inferior parietal and the medial frontal cortex not to be affected until moderate-severe stages of the disease. Cholinergic innervation to the occipital lobe from the nucleus basalis of Meynert was shown to be disrupted in Parkinson’s disease, with increasing cholinergic neuronal loss with the progression of the disease [45].

Several studies have shown that cholinergic innervation of neurons in the occipital cortex influences visual attention and responses [46–48], and it is not surprising therefore that deficient cholinergic input might influence visual processing. The cholinergic activity also has the potential to influence visual processing indirectly, through its actions on the vasculature. Work in mice has demonstrated that donepezil can promote angiogenesis (by increasing HIF-1 alpha and VEGF) [21]. A study in human endothelial cells had similar findings, but only when ACh acted via muscarinic and not nicotinic receptors [49]. It is also known that cholinergic stimulation causes vasodilatation, although in some situations ACh is involved in vasoconstriction [22, 50, 51]. It is therefore possible, but not yet proven, that cholinergic denervation may reduce perfusion of the affected brain areas [52, 53].

Visual hallucinations have also been shown to occur earlier in DLB than in AD [54]. Visual hallucinations are rare in MCI but become more common in all-cause early dementia [55]. One large study based in a specialist early AD clinic found very low rates of VH, and the authors commented that VH seemed to be more prevalent in the later stages of the illness [56], perhaps reflecting cholinergic denervation in areas of the cortex affected later on than the temporal lobe. EEG slowing in AD and visual hallucinations was interpreted as a potential neurophysiological indicator of cholinergic deficiency [57].

Our finding of no between-group difference in ChAT activity is in contrast to the findings of another group, in a smaller study which studied BA18/19 as a single sample. They found ChAT activity to be reduced in BA17 and BA18/19 in both DLB and AD, but with a larger reduction in DLB [17]. Our current study had similar Braak stages and longer disease duration than the previous study, so these factors cannot explain the different results. Our study used a different method for measuring ChAT activity and was also larger (particularly the control group), which may account for the discrepant findings. One explanation for the lack of difference in ChAT activity is that astrocytes are able to express ChAT on demand and this may mask a reduction in neuronal ChAT that might be caused by cholinergic neurodegeneration [36].

Although AChE hydrolyses acetylcholine more quickly than BChE, AChE is inhibited at higher concentrations of ACh. BChE is not and works more efficiently at higher concentrations. Both enzymes need to be measured to assess the changes in cholinergic function [58]. As there was no between-group difference in BChE, our results suggest that any differences in the cholinergic activity are occurring at lower concentrations of the neurotransmitter. Many of the patients would have taken AChE inhibitors, but it would be expected that such inhibition would, if anything, lead to an increase in AChE rather than a decrease [59]. In support of our results, AChE activity was found to be reduced by 27% in a small PET study of patients with DLB and Parkinson’s disease dementia, especially in the medial occipital cortex [60].

A previous study by our group found evidence of chronically reduced oxygenation of primary visual cortex in DLB and evidence that this was due to reduced microvessel density rather than vasoconstriction [18]. This is important as cholinergic innervation of the blood vessels is thought to play a role in regulating cerebral blood flow by causing vasodilatation [22]. In rats, cholinergic projections from the basal forebrain have been shown to release ACh and cause vasodilation when activated [61]. As in our previous study on BA17 (primary visual cortex), MAG was slightly reduced in BA18 and BA19 in the DLB group compared to controls, although the statistical evidence for this was very weak. The MAG:PLP1 ratio was not measured in our previous study. In the present study, VEGF was raised in the DLB group, whereas it was decreased in our previous study. The differences may relate to the differences between the specific areas of the visual cortex that were assessed, but it is also noteworthy that the present study was considerably larger, the DLB and control groups both being doubled in size.

We found no between-group difference in the (very low) α-synuclein field fraction in the occipital cortex but evidence of increased insoluble α-synuclein in the DLB group in both BA18 and BA19. This is entirely in keeping with work by Khundakar et al. and our own group [18, 62]. Khundakar et al. also did not demonstrate any difference between DLB, AD and controls in capillary density in the visual cortex.

Strengths of our present study are the relatively large number of individuals with AD and DLB as well as the size of the control cohort. The assays yielded consistent data, giving us confidence in our results. The main weakness of this study is our reliance on the retrospective collection of data on the presence or absence of visual hallucinations. We measured indirect markers of chronic hypoperfusion, but our previous studies have shown these measures to be reliable markers of hypoperfusion in several diseases and under a wide range of post-mortem conditions. The study was limited to selected visual processing areas in the ventral occipital cortex. Future studies should assess other visual processing areas, including V4–V6. Other brain areas that have been associated with visual hallucinations, including the superior colliculus and the cuneus, should also be considered [63, 64].

## Conclusion

In conclusion, this relatively large post-mortem brain tissue study did not show evidence of more severe α-synuclein pathology or chronic hypoperfusion in the ventral visual processing areas V2 and V3 in AD with visual hallucinations, compared to AD without visual hallucinations or controls. There was also no difference in the ChAT activity. However, AChE activity was reduced in DLB and AD and particularly so in AD with visual hallucinations.

## Additional file

Additional file 1: **Figure S1.** As can be seen, MAG:PLP1 correlated negatively with VEGF, although the relationship was relatively weak. **Figure S2.** ChAT concentration. There was no between-group difference in ChAT concentration in either BA18 (A) or BA19 (B). VH = visual hallucinations. **Figure S3.** ChAT activity. There was no significant between-group difference in ChAT activity in either brain area. **Figure S4.** Neither BChE activity nor concentration differed between the groups in either brain area. There was little relationship between either AChE or BChE activity and protein concentration, suggesting that some of the enzyme present was inactive. **Figure S5.** Examining the relationship between ChAT activity and the activity of cholinergic breakdown enzymes. The cholinergic index in A and B was calculated as ChAT activity/AChE activity + BChE activity. In C and D, the ration of ChAT activity to AChE activity alone is shown. **Figure S6.** Cholinergic markers and disease duration. ChAT activity (A and B), AChE activity (C and D) and BChE activity (E and F) activity did not change significantly with disease duration. **Table S1.** α-Synuclein results. **Table S2.** Comparison between diagnosis in life and the pathological diagnosis made post-mortem. The most common diagnosis listed in the other dementia column was unspecified “dementia” or “senile dementia”. **Table S3.** Further details on medical history for the individuals whose donated tissue was used in this study. The “other neurological diagnoses” included epilepsy late on in dementia (3 people), minor head injuries, vascular Parkinsonism (1 person) and possible SLE (1 person). It was apparent that some individuals were treated with antipsychotics to control distress at the end of their lives, rather than to treat psychotic symptoms. (DOCX 772 kb)

## Data Availability

Unfortunately, the fresh tissue samples used in this paper are not available. The raw data are available on request, subject to the conditions of the ethical approval.

## Abbreviations

AChE: Acetylcholinesterase
AD: Alzheimer’s disease
BA17: Brodmann area 17
BA18: Brodmann area 18
BA19: Brodmann area 19
BChE: Butyrlcholinesterase
ChAT: Cholineacetyltransferase
DLB: Dementia with Lewy bodies
DSM-IV: Diagnostic and statistical manual of mental disorders, 4th edition
ELISA: Enzyme-linked immunosorbent assay
MAG: Myelin-associated glycoprotein
NP-40: 4-Nonylphenyl poly (ethylene glycol)
PBS: Phosphate-buffered saline
PET: Positron emission tomography
PLP: Phospholipoprotein 1
SDS: Sodium dodecyl sulphate
SPECT: Single-photon emission computed tomography
TBS: Tris-buffered saline
VEGF: Vascular endothelial growth factor
VH: Visual hallucinations
vWF: von Willebrand Factor
