# Supplementary material for: Visual hallucinations in Alzheimer's disease do not seem to be associated with chronic hypoperfusion of to visual processing areas V2 and V3 but may be associated with reduced cholinergic input to these areas
Source: Alzheimers Res Ther. 2019 Sep 12;11:80. doi: 10.1186/s13195-019-0519-7 (PMC6740037; doi:10.1186/s13195-019-0519-7)
Supplement: Supplementary file 1 — Figure S1. As can be seen, MAG:PLP1 correlated negatively with VEGF, although the relationship was relatively weak. Figure S2. ChAT concentration. There was no between-group difference in ChAT concentration in either BA18 (A) or BA19 (B). VH = visual hallucinations. Figure S3. ChAT activity. There was no significant between-group difference in ChAT activity in either brain area. Figure S4. Neither BChE activity nor concentration differed between the groups in either brain area. There was little relationship between either AChE or BChE activity and protein concentration, suggesting that some of the enzyme present was inactive. Figure S5. Examining the relationship between ChAT activity and the activity of cholinergic breakdown enzymes. The cholinergic index in A and B was calculated as ChAT activity/AChE activity + BChE activity. In C and D, the ration of ChAT activity to AChE activity alone is shown. Figure S6. Cholinergic markers and disease duration. ChAT activity (A and B), AChE activity (C and D) and BChE activity (E and F) activity did not change significantly with disease duration. Table S1. α-Synuclein results. Table S2. Comparison between diagnosis in life and the pathological diagnosis made post-mortem. The most common diagnosis listed in the other dementia column was unspecified “dementia” or “senile dementia”. Table S3. Further details on medical history for the individuals whose donated tissue was used in this study. The “other neurological diagnoses” included epilepsy late on in dementia (3 people), minor head injuries, vascular Parkinsonism (1 person) and possible SLE (1 person). It was apparent that some individuals were treated with antipsychotics to control distress at the end of their lives, rather than to treat psychotic symptoms. (DOCX 772 kb) [file 13195_2019_519_MOESM1_ESM.docx]

**Supplementary Information**

Figure S1: As can be seen MAG:PLP1 correlated negatively with VEGF, although the relationship was relatively weak.





Figure S2: ChAT concentration. There was no between-group difference in ChAT concentration in either BA18 (A) or BA19 (B). VH = visual hallucinations.

**

**

Figure S3: ChAT activity. There was no significant between-group difference in ChAT activity in either brain area.

**

**

Figure S4: Neither BChE activity nor concentration differed between groups in either brain area. There was little relationship between either AChE or BChE activity and protein concentration, suggesting that some of the enzyme present was inactive.





Figure S5: Examining the relationship between ChAT activity and the activity of cholinergic breakdown enzymes. The cholinergic index in A & B was calculated as ChAT activity/AChE activity + BChE activity. In C & D the ration of ChAT activity to AChE activity alone is shown.





Figure S6: Cholinergic markers and disease duration. ChAT activity (A & B), AChE activity (C & D) and BChE activity (E & F) activity did not change significantly with disease duration.

**

**

Table S1: α-Synuclein results

|  | **AD with visual hallucinations**  **n = 23** | | **AD without visual hallucinations**  **n = 19** | | **Dementia with Lewy bodies**  **n = 19** | | **Controls**  **n = 36** | | **Statistical Evidence** |
| --- | --- | --- | --- | --- | --- | --- | --- | --- | --- |
|  | **Mean** | **SD** | **Mean** | **SD** | **Mean** | **SD** | **Mean** | **SD** |  |
| Insoluble α-synuclein BA18 µg/mg | 0.654 | 0.445 | 0.547 | 0.272 | 0.981 | 0.472 | 0.861 | 0.505 | Kruskal Wallis  Χ^2^ = 12.48, p = 0.006 |
| Soluble α-synuclein BA18 µg/mg | 5.21 | 2.592 | 4.985 | 2.926 | 6.932 | 3.094 | 5.368 | 2.229 | ANOVA p = 0.090 |
| Insoluble α-synuclein BA19 µg/mg | 1.324 | 0.513 | 1.557 | 0.456 | 1.807 | 0.531 | 1.633 | 0.496 | Kruskal Wallis  Χ^2^ = 10.197, p = 0.017 |
| Soluble α-synuclein BA19 µg/mg | 1.599 | 0.919 | 1.741 | 1.467 | 2.882 | 2.038 | 2.049 | 1.491 | ANOVA p = 0.032 |
| α-synuclein field fraction BA18 | 0.017 | 0.029 | 0.009 | 0.015 | 0.017 | 0.035 | 0.03 | 0.061 | Kruskal Wallis, Χ^2^ = 1.5,  p = 0.683 |
| α-synuclein field fraction BA19 | 0.015 | 0.026 | 0.008 | 0.014 | 0.014 | 0.027 | 0.031 | 0.05 | Kruskal Wallis, Χ^2^ = 1.62, p = 0.654 |

| **Diagnosis during life** | **AD with visual hallucinations**  **n = 23** | **AD without visual hallucinations**  **n = 19** | **Dementia with Lewy bodies**  **n = 19** | **Controls**  **n = 36** |
| --- | --- | --- | --- | --- |
| **Alzheimer’s disease** | 9 | 13 | 5 | 0 |
| **Mixed dementia** | 1 | 2 | 0 | 0 |
| **Dementia with Lewy bodies** | 4 | 0 | 7 | 0 |
| **Frontotemporal dementia** | 1 | 0 | 0 | 0 |
| **Vascular dementia** | 3 | 1 | 3 | 0 |
| **Other dementia** | 5 | 3 | 4 | 0 |
| **Cognitively intact** | 0 | 0 | 0 | 36 |

Table S2: Comparison between diagnosis in life and the pathological diagnosis made post-mortem. The most common diagnosis listed in the other dementia column was unspecified “dementia” or “senile dementia”.

Table S3: Further details on medical history for the individuals whose donated tissue was used in this study. The “other neurological diagnoses” included epilepsy late on in dementia (3 people), minor head injuries, vascular Parkinsonism (1 person) and possible SLE (1 person). It was apparent that some individuals were treated with antipsychotics to control distress at the end of their lives, rather than to treat psychotic symptoms.

|  | **AD with visual hallucinations**  **n = 23** | **AD without visual hallucinations**  **n = 19** | **Dementia with Lewy bodies**  **n = 19** | **Controls**  **n = 36** |
| --- | --- | --- | --- | --- |
| **Neurological history**  None  CVA/TIA  SDH  Migraine  Other  Epilepsy pre dementia | 13  4  0  1  5  0 | 8  6  1  0  3  1 | 10  6  0  1  2  0 | 19  10  1  0  2  1 |
| **Psychiatric history**  None  Psychotic disorder  Anxiety  Depression  Mixed anxiety/depression  Anxiety/depression only in dementia  Alcohol excess  Other | 11  0  4  5  1  1  0  1 | 9  0  2  2  0  5  1  0 | 8  0  1  2  3  3  1  1 | 21  0  0  9  3  0  0  0 |
| **Number treated with anticholinesterase inhibitors** | 17 | 14 | 11 | 0/33 |
| **Antidepressant Rx**  None  SSRI  NaSSA  SNRI  TCA  Multiple classes  MAOI | 9  8  1  0  2  3  0 | 10  6  0  2  1  3  0 | 9  5  1  0  1  0  1 | 24  6  0  0  0  2  0 |
| **Number treated with antipsychotics** | 12 | 4 | 7 | 3/30 |
| **Other treatment received**  Memantine  Pregabalin  Other | 8  0  1 | 7  0  1 | 1  0  0 | 0  2  1 |
| **Last MMSE Score** | 12.8 (SD 7.1) | 14.7 (SD 7.0) | 12.3 (SD 7.5) | 27.6 (SD 2.3) |
